# Crystal structure and Hirshfeld surface analysis of 1-(4-chloro­phen­yl)-2-{[5-(4-chloro­phen­yl)-1,3,4-oxa­diazol-2-yl]sulfan­yl}ethanone

**DOI:** 10.1107/S2056989017003978

**Published:** 2017-03-17

**Authors:** Rajesh Kumar, Shafqat Hussain, Khalid M. Khan, Shahnaz Perveen, Sammer Yousuf

**Affiliations:** a H. E. J. Research Institute of Chemistry, International Center for Chemical and Biological Sciences, University of Karachi, Karachi-75270, Pakistan; b Karakoram International University, Gilgit, Pakistan; c PCSIR Laboratories Complex, Karachi, Pakistan

**Keywords:** oxadizole, chloro­phen­yl, X-ray structure, Hirshfeld surface analysis, crystal structure

## Abstract

In the title compound, C_16_H_10_Cl_2_N_2_O_2_S, the dihedral angles formed by the chloro-substituted benzene rings with the central oxa­diazole ring are 6.54 (9) and 6.94 (8)°. In the crystal, C—H⋯N hydrogen bonding links the mol­ecules into undulating ribbons running parallel to the *b* axis. Hirshfeld surface analysis indicates that the most important contributions for the crystal packing are the H⋯C (18%), H⋯H (17%), H⋯Cl (16.6%), H⋯O (10.4%), H⋯N (8.9%) and H⋯S (5.9%) inter­actions.

## Abstract 2

The title heterocyclic compound is contains an oxadizole and two chloro-substituted phenyl rings. In the crystal, C—H⋯N hydrogen bonding links the mol­ecules into undulating ribbons parallel to the *b* axis. Hirshfeld surface analysis indicates that the most important contributions for the crystal packing are the H⋯C (18%), H⋯H (17%), H⋯Cl (16.6%), H⋯O (10.4%), H⋯N (8.9%) and H⋯S (5.9%) inter­actions.

## Chemical context   

Heterocyclic compounds are well known for their applications in agriculture (Jakobi *et al.*, 1999 ▸) and for the synthesis of pharmaceuticals (Vitaku *et al.*, 2014 ▸). The broad range of biological activities of heterocyclic compounds has always fascinated chemists and the literature reveals many approaches to synthesize and derivatize libraries of heterocyclic compounds (Khan *et al.*, 2011 ▸; Chohan *et al.*, 2006 ▸; Khan *et al.*, 2005 ▸). The wide range of applications and biological activities of this class of compounds is due to the presence of heteroatoms (N, O, S) in the mol­ecule (Kashtoh *et al.*, 2014 ▸). Oxa­diazo­les are among the most widely studied moieties of organic chemistry due to their many important chemical and biological properties including anti­mycobacterial (Jha *et al.*, 2009 ▸), anti­oxidant (Fadda *et al.*, 2011 ▸), anti­cancer (Zhang *et al.*, 2011 ▸), anti­tumor (Loetchutinat *et al.*, 2003 ▸), anti­microbial (Şahin *et al.*, 2002 ▸), anti­fungal (Zou *et al.*, 2002 ▸), anti-inflammatory (Palaska *et al.*, 2002 ▸) and hypotensive (Tyagi & Kumar, 2002 ▸) activities.
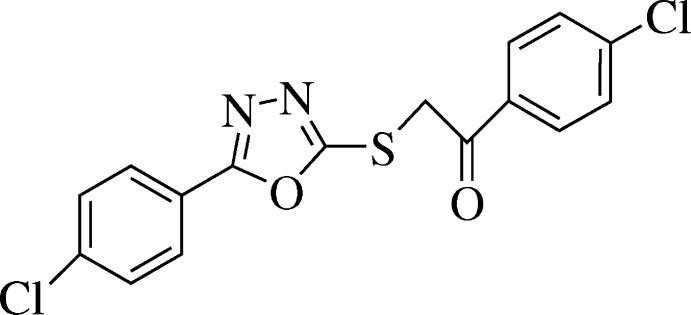


## Structural commentary   

**Figure 1:**
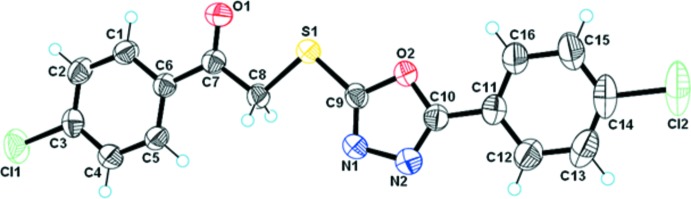
The mol­ecular structure of the title compound with displacement ellipsoids drawn at 30% probability level.

The title compound (Fig. 1 ▸) is an oxa­diazole derivative containing two chloro­phenyl substituents attached to a central oxa­diazole thio­ethanone unit. The C1–C6 and C11–C16 phenyl rings form dihedral angles of 6.54 (9) and 6.94 (8)°, respectively, with the oxa­diazole ring. The dihedral angle between the oxa­diazole ring and the mean plane through the S1/O1/C7–C8 fragment is 10.75 (8)°. Bond lengths and angles are not unusual.

## Supra­molecular features   

**Figure 2:**
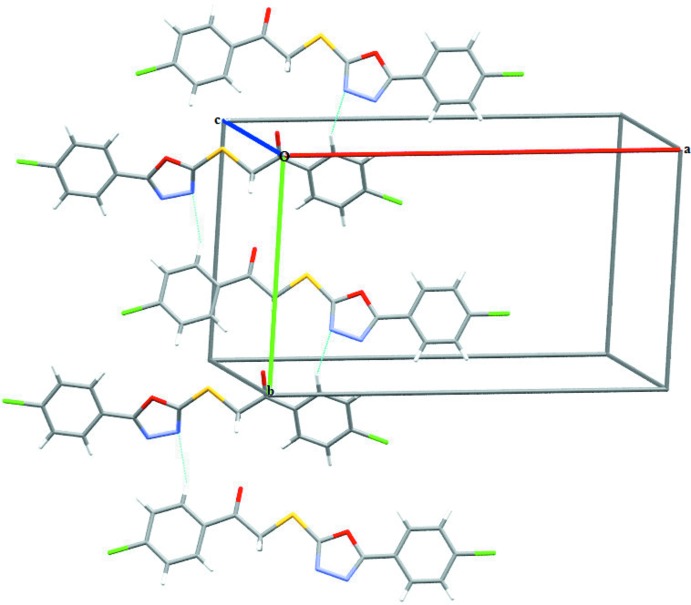
Partial crystal packing of the title compound showing the formation of a undulating ribbon parallel to the *b* axis through C—H⋯N hydrogen bonds (dashed lines).

In the crystal, mol­ecules are connected by C—H⋯N hydrogen inter­actions, forming undulating ribbons parallel to the *b* axis (Table 1 ▸, Fig. 2 ▸). The importance of these inter­actions in stabilizing the crystal structure may be determined by comparison with those found in similar related compounds. For instance, in the crystal structure of 2-{5-[(1*H*-1,2,4-triazol-1-yl)meth­yl]-1,3,4-oxa­diazol-2-yl­thio}-1-(2,4-di­chloro­phen­yl)ethanone (Xu *et al.*, 2005 ▸) mol­ecules are linked into chains *via* C—H⋯N hydrogen bonds having H⋯N separations of 2.48 Å. and C—H⋯C inter­actions having H⋯N distances of 2.41 Å. Similarly, in the crystal structure of 1,3-*bis*{[5-(pyridin-2-yl)-1,3,4-oxa­diazol-2-yl]sulfan­yl}propan-2-one (Xia *et al.*, 2011 ▸), two oxa­diazole rings are present and form inter­molecular hydrogen bonds of the type C—H⋯N with distances of 2.51 and 2.54 Å, respectively. Moreover, in the structure of the latter compound, further stabilization of the crystal structure is provided by π–π inter­actions involving the pyridyl and oxa­diazole rings with centroid-to-centroid distances of 3.883 Å.

## Hirshfeld surface analysis   

**Figure 3:**
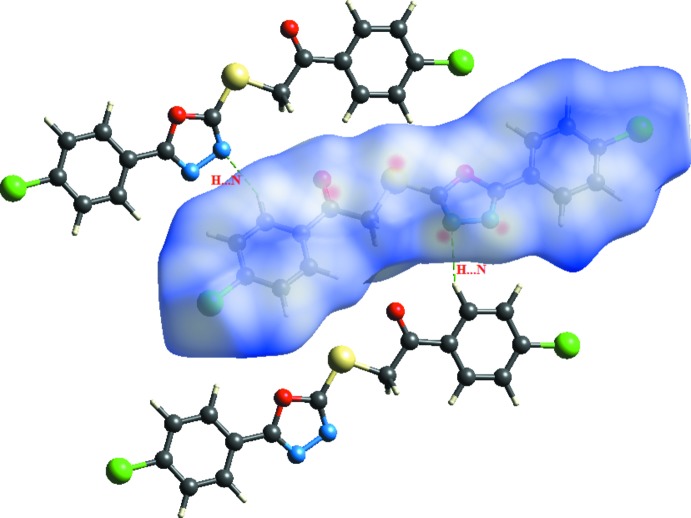
*d*_norm_ mapped on the Hirshfeld surface, visualizing the inter­molecular contacts of the title compound. Dotted lines indicate hydrogen bonds.

**Figure 4:**
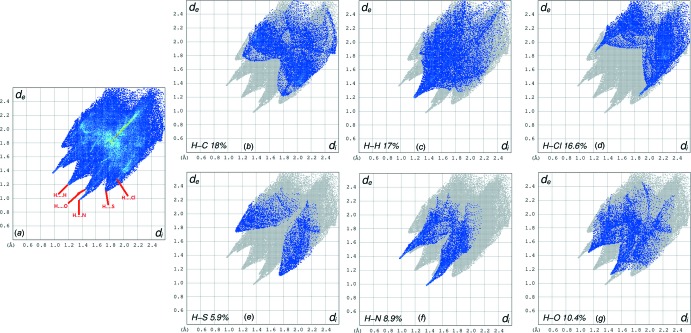
Hirshfeld surface two-dimensional fingerprint plot for the title compound (*a*) showing the: (*b*) H⋯C, (*c*) H⋯H, (*d*) H⋯Cl, (*e*) H⋯S, (*f*) H⋯N and (*g*) H⋯O inter­actions. The outline of the full fingerprint plots is shown in gray. *d*_i_ (*x* axis) and *d*_e_ (*y* axis) are the closest inter­nal and external distance (values in Å) from a given point on the Hirshfeld surface contacts.

The Hirshfeld surface analysis (Spackman & Jayatilaka, 2009 ▸) of the crystal structure suggests that the contribution to the crystal packing of the H⋯N inter­action is 8.9% (Fig. 3 ▸). Other important inter­actions based upon the percentages are H⋯H (17%), H⋯O (10.4%), H⋯C (18%), H⋯S (5.9%) and H⋯Cl (16.6%). These inter­actions, however, were not found to be involved in hydrogen bonding, as observed for the H⋯N contribution (Fig. 4 ▸). The Hirshfeld surface diagram shows the location of atoms with the potential to form hydrogen bonds. These inter­actions are represented in two-dimensional fingerprint plots (Fig. 4 ▸), in which the cyan dots indicate the percentage of the inter­action over the total Hirshfeld surface.

## Synthesis and crystallization   

The title compound was synthesized by the procdure reported by Kashtoh *et al.* (2014 ▸). 4-Chloro-1,3,4-oxa­diazole-2-thiol (212 mg,1 mmol) and triethyl amine (0.1 mL) were taken in ethanol (10 mL) and stirred for 10 min. 2-Bromo-4′-chloro­aceto­phenone (232 mg, 1 mmol) was then added slowly into the mixture and refluxed, while progress of the reaction was monitored by TLC. After completion of the reaction, the precipitate was filtered and washed with ethanol. The precipitate was crystallized from methanol to give the title compound in 344 mg, 94% yield.

## Refinement   

Crystal data, data collection and structure refinement details are summarized in (Table 2 ▸). H atoms were located in a difference-Fourier map, but were positioned with idealized geometry and refined with C—H = 0.93–0.97 Å, and with *U*_iso_(H) = 1.2*U*_eq_(C).

## Supplementary Material

Crystal structure: contains datablock(s) global, I. DOI: 10.1107/S2056989017003978/rz5206sup1.cif

Structure factors: contains datablock(s) I. DOI: 10.1107/S2056989017003978/rz5206Isup2.hkl

Supporting information file. DOI: 10.1107/S2056989017003978/rz5206Isup3.cml

CCDC reference: 1537363

Additional supporting information:  crystallographic information; 3D view; checkCIF report

## Figures and Tables

**Table 1:** Hydrogen-bond geometry (Å, °)

*D*—H⋯*A*	*D*—H	H⋯*A*	*D*⋯*A*	*D*—H⋯*A*
C1—H1*B*⋯N1^i^	0.93	2.48	3.353 (3)	157

Symmetry code: (i) 
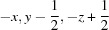
.

**Table 2:** Experimental details

Crystal data	
Chemical formula	C_16_H_10_Cl_2_N_2_O_2_S
*M*_r_	365.22
Crystal system, space group	Monoclinic, *P*2_1_/*c*
Temperature (K)	273
*a*, *b*, *c* (Å)	19.1513 (7), 11.1589 (4), 7.5071 (3)
β (°)	92.088 (1)
*V* (Å^3^)	1603.26 (10)
*Z*	4
Radiation type	Mo *K*α
μ (mm^−1^)	0.55
Crystal size (mm)	0.47 × 0.39 × 0.11

Data collection	
Diffractometer	Bruker *SMART* *APEX* CCD area-detector
Absorption correction	Multi-scan (*SADABS*; Bruker, 2000 ▸)
*T*_min_, *T*_max_	0.784, 0.945
No. of measured, independent and observed [*I* > 2σ(*I*)] reflections	11526, 3762, 3058
*R*_int_	0.022
(sin θ/λ)_max_ (Å^−1^)	0.667

Refinement	
*R*[*F* ^2^ > 2σ(*F* ^2^)], *wR*(*F* ^2^), *S*	0.045, 0.132, 1.12
No. of reflections	3762
No. of parameters	208
H-atom treatment	H-atom parameters constrained
Δρ_max_, Δρ_min_ (e Å^−3^)	0.34, −0.26

Computer programs: *SMART* and *SAINT* (Bruker, 2000 ▸), *SHELXS97*, *SHELXL97* and *SHELXTL* (Sheldrick, 2008 ▸), *PARST* (Nardelli, 1995 ▸) and *PLATON* (Spek, 2009 ▸).

## supplementary crystallographic information

### Crystal data

**Table:**

C_16_H_10_Cl_2_N_2_O_2_S	*F*(000) = 744
*M**_r_* = 365.22	*D*_x_ = 1.513 Mg m^−^^3^
Monoclinic, *P*2_1_/*c*	Mo *K*α radiation, λ = 0.71073 Å
*a* = 19.1513 (7) Å	Cell parameters from 3559 reflections
*b* = 11.1589 (4) Å	θ = 3.2–27.7°
*c* = 7.5071 (3) Å	µ = 0.55 mm^−^^1^
β = 92.088 (1)°	*T* = 273 K
*V* = 1603.26 (10) Å^3^	Block, colorless
*Z* = 4	0.47 × 0.39 × 0.11 mm

### Data collection

**Table:**

Bruker SMART APEX CCD area-detector diffractometer	3762 independent reflections
Radiation source: fine-focus sealed tube	3058 reflections with *I* > 2σ(*I*)
Graphite monochromator	*R*_int_ = 0.022
ω scan	θ_max_ = 28.3°, θ_min_ = 1.1°
Absorption correction: multi-scan (SADABS; Bruker, 2000)	*h* = −25→25
*T*_min_ = 0.784, *T*_max_ = 0.945	*k* = −13→14
11526 measured reflections	*l* = −9→9

### Refinement

**Table:**

Refinement on *F*^2^	Primary atom site location: structure-invariant direct methods
Least-squares matrix: full	Secondary atom site location: difference Fourier map
*R*[*F*^2^ > 2σ(*F*^2^)] = 0.045	Hydrogen site location: inferred from neighbouring sites
*wR*(*F*^2^) = 0.132	H-atom parameters constrained
*S* = 1.12	*w* = 1/[σ^2^(*F*_o_^2^) + (0.069*P*)^2^ + 0.2639*P*] where *P* = (*F*_o_^2^ + 2*F*_c_^2^)/3
3762 reflections	(Δ/σ)_max_ < 0.001
208 parameters	Δρ_max_ = 0.34 e Å^−^^3^
0 restraints	Δρ_min_ = −0.26 e Å^−^^3^

### Special details

**Table:**

Geometry. All esds (except the esd in the dihedral angle between two l.s. planes) are estimated using the full covariance matrix. The cell esds are taken into account individually in the estimation of esds in distances, angles and torsion angles; correlations between esds in cell parameters are only used when they are defined by crystal symmetry. An approximate (isotropic) treatment of cell esds is used for estimating esds involving l.s. planes.
Refinement. Refinement of F^2^ against ALL reflections. The weighted R-factor wR and goodness of fit S are based on F^2^, conventional R-factors R are based on F, with F set to zero for negative F^2^. The threshold expression of F^2^ > 2sigma(F^2^) is used only for calculating R-factors(gt) etc. and is not relevant to the choice of reflections for refinement. R-factors based on F^2^ are statistically about twice as large as those based on F, and R- factors based on ALL data will be even larger.

### Fractional atomic coordinates and isotropic or equivalent isotropic displacement parameters (Å^2^)

**Table:**

	*x*	*y*	*z*	*U*_iso_*/*U*_eq_	
Cl1	−0.31166 (3)	0.71345 (7)	0.43607 (10)	0.0711 (2)	
Cl2	0.54160 (4)	0.62796 (12)	−0.30547 (16)	0.1258 (4)	
S1	0.09911 (2)	0.52100 (4)	0.14177 (7)	0.04411 (16)	
O1	−0.01151 (8)	0.43467 (13)	0.3026 (2)	0.0556 (4)	
O2	0.21861 (7)	0.57741 (12)	0.00900 (19)	0.0427 (3)	
N1	0.14210 (9)	0.72473 (14)	−0.0287 (2)	0.0457 (4)	
N2	0.20649 (9)	0.75906 (16)	−0.1028 (3)	0.0508 (4)	
C1	−0.14356 (11)	0.50990 (18)	0.3877 (3)	0.0448 (5)	
H1B	−0.1303	0.4313	0.4130	0.054*	
C2	−0.20957 (11)	0.54753 (19)	0.4258 (3)	0.0494 (5)	
H2B	−0.2410	0.4953	0.4766	0.059*	
C3	−0.22851 (10)	0.6646 (2)	0.3873 (3)	0.0463 (5)	
C4	−0.18228 (10)	0.74359 (19)	0.3131 (3)	0.0471 (5)	
H4A	−0.1958	0.8222	0.2889	0.057*	
C5	−0.11620 (10)	0.70548 (17)	0.2752 (3)	0.0428 (4)	
H5A	−0.0849	0.7583	0.2250	0.051*	
C6	−0.09600 (9)	0.58753 (16)	0.3118 (3)	0.0375 (4)	
C7	−0.02636 (10)	0.53891 (16)	0.2705 (3)	0.0394 (4)	
C8	0.02691 (9)	0.61879 (16)	0.1858 (3)	0.0410 (4)	
H8A	0.0415	0.6828	0.2663	0.049*	
H8B	0.0079	0.6537	0.0761	0.049*	
C9	0.15279 (9)	0.61954 (16)	0.0337 (3)	0.0385 (4)	
C10	0.24856 (10)	0.67088 (18)	−0.0775 (3)	0.0419 (4)	
C11	0.32086 (10)	0.6589 (2)	−0.1300 (3)	0.0466 (5)	
C12	0.35671 (13)	0.7597 (2)	−0.1857 (3)	0.0611 (6)	
H12A	0.3347	0.8340	−0.1876	0.073*	
C13	0.42443 (14)	0.7499 (3)	−0.2380 (4)	0.0743 (8)	
H13A	0.4485	0.8174	−0.2747	0.089*	
C14	0.45624 (12)	0.6400 (3)	−0.2356 (4)	0.0760 (8)	
C15	0.42210 (13)	0.5394 (3)	−0.1805 (5)	0.0819 (9)	
H15A	0.4444	0.4654	−0.1793	0.098*	
C16	0.35398 (12)	0.5494 (2)	−0.1265 (4)	0.0643 (7)	
H16A	0.3305	0.4818	−0.0877	0.077*	

### Atomic displacement parameters (Å^2^)

**Table:**

	*U*^11^	*U*^22^	*U*^33^	*U*^12^	*U*^13^	*U*^23^
Cl1	0.0415 (3)	0.0839 (5)	0.0889 (5)	0.0064 (3)	0.0182 (3)	−0.0073 (3)
Cl2	0.0440 (4)	0.1827 (11)	0.1531 (10)	−0.0066 (5)	0.0351 (5)	0.0047 (8)
S1	0.0393 (3)	0.0369 (3)	0.0567 (4)	0.00321 (18)	0.0088 (2)	0.0004 (2)
O1	0.0519 (8)	0.0395 (8)	0.0761 (11)	0.0055 (6)	0.0118 (7)	0.0083 (7)
O2	0.0347 (6)	0.0408 (7)	0.0531 (9)	0.0030 (5)	0.0068 (6)	0.0007 (6)
N1	0.0402 (8)	0.0399 (9)	0.0575 (11)	0.0064 (7)	0.0061 (7)	0.0015 (7)
N2	0.0464 (9)	0.0448 (9)	0.0615 (12)	0.0013 (8)	0.0073 (8)	0.0058 (8)
C1	0.0474 (11)	0.0396 (10)	0.0477 (12)	−0.0031 (8)	0.0055 (9)	0.0040 (8)
C2	0.0458 (11)	0.0505 (12)	0.0527 (13)	−0.0098 (9)	0.0122 (9)	0.0030 (9)
C3	0.0349 (9)	0.0569 (12)	0.0472 (12)	−0.0011 (8)	0.0050 (8)	−0.0066 (9)
C4	0.0446 (11)	0.0421 (10)	0.0550 (13)	0.0043 (8)	0.0053 (9)	−0.0002 (9)
C5	0.0415 (10)	0.0385 (10)	0.0488 (12)	−0.0034 (7)	0.0067 (8)	0.0025 (8)
C6	0.0376 (9)	0.0369 (9)	0.0380 (11)	−0.0020 (7)	0.0025 (7)	−0.0035 (7)
C7	0.0407 (9)	0.0384 (10)	0.0393 (11)	−0.0018 (7)	0.0024 (8)	−0.0039 (7)
C8	0.0349 (9)	0.0374 (10)	0.0511 (12)	−0.0001 (7)	0.0062 (8)	−0.0050 (8)
C9	0.0341 (9)	0.0387 (9)	0.0428 (11)	0.0023 (7)	0.0020 (7)	−0.0067 (8)
C10	0.0387 (9)	0.0428 (10)	0.0441 (12)	−0.0009 (8)	0.0022 (8)	−0.0023 (8)
C11	0.0380 (10)	0.0553 (12)	0.0466 (12)	−0.0055 (8)	0.0017 (8)	−0.0023 (9)
C12	0.0542 (13)	0.0643 (14)	0.0652 (16)	−0.0073 (11)	0.0064 (11)	0.0097 (12)
C13	0.0563 (15)	0.094 (2)	0.0731 (19)	−0.0240 (15)	0.0099 (12)	0.0140 (15)
C14	0.0358 (11)	0.115 (2)	0.0776 (19)	−0.0088 (13)	0.0123 (11)	−0.0057 (16)
C15	0.0443 (13)	0.0813 (19)	0.121 (3)	0.0050 (12)	0.0181 (14)	−0.0089 (17)
C16	0.0429 (11)	0.0570 (13)	0.0939 (19)	−0.0010 (10)	0.0144 (12)	−0.0044 (13)

### Geometric parameters (Å, º)

**Table:**

Cl1—C3	1.735 (2)	C5—C6	1.396 (3)
Cl2—C14	1.740 (2)	C5—H5A	0.9300
S1—C9	1.7279 (19)	C6—C7	1.483 (2)
S1—C8	1.8014 (18)	C7—C8	1.512 (2)
O1—C7	1.219 (2)	C8—H8A	0.9700
O2—C9	1.364 (2)	C8—H8B	0.9700
O2—C10	1.366 (2)	C10—C11	1.459 (3)
N1—C9	1.277 (2)	C11—C16	1.376 (3)
N1—N2	1.424 (2)	C11—C12	1.390 (3)
N2—C10	1.281 (3)	C12—C13	1.373 (3)
C1—C2	1.372 (3)	C12—H12A	0.9300
C1—C6	1.394 (3)	C13—C14	1.369 (4)
C1—H1B	0.9300	C13—H13A	0.9300
C2—C3	1.384 (3)	C14—C15	1.370 (4)
C2—H2B	0.9300	C15—C16	1.385 (3)
C3—C4	1.381 (3)	C15—H15A	0.9300
C4—C5	1.375 (3)	C16—H16A	0.9300
C4—H4A	0.9300		
			
C9—S1—C8	100.05 (9)	C7—C8—H8B	110.8
C9—O2—C10	101.97 (14)	S1—C8—H8B	110.8
C9—N1—N2	105.12 (15)	H8A—C8—H8B	108.9
C10—N2—N1	106.51 (16)	N1—C9—O2	113.80 (16)
C2—C1—C6	121.04 (18)	N1—C9—S1	131.81 (14)
C2—C1—H1B	119.5	O2—C9—S1	114.39 (13)
C6—C1—H1B	119.5	N2—C10—O2	112.59 (17)
C1—C2—C3	118.81 (18)	N2—C10—C11	128.91 (19)
C1—C2—H2B	120.6	O2—C10—C11	118.50 (17)
C3—C2—H2B	120.6	C16—C11—C12	119.5 (2)
C4—C3—C2	121.32 (18)	C16—C11—C10	121.17 (19)
C4—C3—Cl1	119.47 (17)	C12—C11—C10	119.4 (2)
C2—C3—Cl1	119.20 (16)	C13—C12—C11	120.2 (3)
C5—C4—C3	119.66 (19)	C13—C12—H12A	119.9
C5—C4—H4A	120.2	C11—C12—H12A	119.9
C3—C4—H4A	120.2	C14—C13—C12	119.5 (2)
C4—C5—C6	120.09 (18)	C14—C13—H13A	120.3
C4—C5—H5A	120.0	C12—C13—H13A	120.3
C6—C5—H5A	120.0	C13—C14—C15	121.4 (2)
C1—C6—C5	119.08 (17)	C13—C14—Cl2	119.2 (2)
C1—C6—C7	117.65 (17)	C15—C14—Cl2	119.4 (2)
C5—C6—C7	123.26 (17)	C14—C15—C16	119.2 (3)
O1—C7—C6	120.85 (17)	C14—C15—H15A	120.4
O1—C7—C8	119.29 (17)	C16—C15—H15A	120.4
C6—C7—C8	119.85 (16)	C11—C16—C15	120.3 (2)
C7—C8—S1	104.72 (12)	C11—C16—H16A	119.9
C7—C8—H8A	110.8	C15—C16—H16A	119.9
S1—C8—H8A	110.8		
			
C9—N1—N2—C10	0.2 (2)	C10—O2—C9—S1	179.80 (13)
C6—C1—C2—C3	0.1 (3)	C8—S1—C9—N1	−11.3 (2)
C1—C2—C3—C4	−0.5 (3)	C8—S1—C9—O2	169.15 (14)
C1—C2—C3—Cl1	−179.61 (17)	N1—N2—C10—O2	−0.1 (2)
C2—C3—C4—C5	0.5 (3)	N1—N2—C10—C11	179.0 (2)
Cl1—C3—C4—C5	179.62 (16)	C9—O2—C10—N2	0.0 (2)
C3—C4—C5—C6	−0.1 (3)	C9—O2—C10—C11	−179.28 (17)
C2—C1—C6—C5	0.3 (3)	N2—C10—C11—C16	−166.0 (2)
C2—C1—C6—C7	−178.44 (19)	O2—C10—C11—C16	13.1 (3)
C4—C5—C6—C1	−0.3 (3)	N2—C10—C11—C12	13.5 (4)
C4—C5—C6—C7	178.38 (18)	O2—C10—C11—C12	−167.4 (2)
C1—C6—C7—O1	−0.2 (3)	C16—C11—C12—C13	0.5 (4)
C5—C6—C7—O1	−178.9 (2)	C10—C11—C12—C13	−179.1 (2)
C1—C6—C7—C8	179.18 (17)	C11—C12—C13—C14	0.3 (4)
C5—C6—C7—C8	0.5 (3)	C12—C13—C14—C15	−0.6 (5)
O1—C7—C8—S1	4.4 (2)	C12—C13—C14—Cl2	179.0 (2)
C6—C7—C8—S1	−174.98 (14)	C13—C14—C15—C16	0.1 (5)
C9—S1—C8—C7	176.28 (13)	Cl2—C14—C15—C16	−179.4 (2)
N2—N1—C9—O2	−0.2 (2)	C12—C11—C16—C15	−0.9 (4)
N2—N1—C9—S1	−179.78 (16)	C10—C11—C16—C15	178.6 (2)
C10—O2—C9—N1	0.2 (2)	C14—C15—C16—C11	0.6 (5)

### Hydrogen-bond geometry (Å, º)

**Table:**

*D*—H···*A*	*D*—H	H···*A*	*D*···*A*	*D*—H···*A*
C1—H1*B*···N1^i^	0.93	2.48	3.353 (3)	157

Symmetry code: (i) −*x*, *y*−1/2, −*z*+1/2.
